# Virtual Drug Repositioning as a Tool to Identify Natural Small Molecules That Synergize with Lumacaftor in F508del-CFTR Binding and Rescuing

**DOI:** 10.3390/ijms232012274

**Published:** 2022-10-14

**Authors:** Paola Fossa, Matteo Uggeri, Alessandro Orro, Chiara Urbinati, Alessandro Rondina, Maria Milanesi, Nicoletta Pedemonte, Emanuela Pesce, Rita Padoan, Robert C. Ford, Xin Meng, Marco Rusnati, Pasqualina D’Ursi

**Affiliations:** 1 Department of Pharmacy, Section of Medicinal Chemistry, School of Medical and Pharmaceutical Sciences, University of Genoa, 16132 Genoa, Italy; 2 Institute for Biomedical Technologies, National Research Council (ITB-CNR), 20054 Segrate, Italy; 3 Department of Molecular and Translational Medicine, University of Brescia, 25123 Brescia, Italy; 4 UOC Genetica Medica, IRCCS Istituto Giannina Gaslini, 16147 Genova, Italy; 5 Department of Pediatrics, Regional Support Centre for Cystic Fibrosis, Children’s Hospital—ASST Spedali Civili, University of Brescia, 25123 Brescia, Italy; 6 Faculty of Biology, Medicine and Health, University of Manchester, Manchester M13 9PL, UK; 7 Cellular Degradation Systems Laboratory, The Francis Crick Institute, London NW1 1AT, UK

**Keywords:** cystic fibrosis, F508del-CFTR, lumacaftor, nicotinamide, drug repositioning, molecular docking and dynamics, surface plasmon resonance

## Abstract

Cystic fibrosis is a hereditary disease mainly caused by the deletion of the Phe 508 (F508del) of the cystic fibrosis transmembrane conductance regulator (CFTR) protein that is thus withheld in the endoplasmic reticulum and rapidly degraded by the ubiquitin/proteasome system. Cystic fibrosis remains a potentially fatal disease, but it has become treatable as a chronic condition due to some CFTR-rescuing drugs that, when used in combination, increase in their therapeutic effect due to a synergic action. Also, dietary supplementation of natural compounds in combination with approved drugs could represent a promising strategy to further alleviate cystic fibrosis symptoms. On these bases, we screened by in silico drug repositioning 846 small synthetic or natural compounds from the AIFA database to evaluate their capacity to interact with the highly druggable lumacaftor binding site of F508del-CFTR. Among the identified hits, nicotinamide (NAM) was predicted to accommodate into the lumacaftor binding region of F508del-CFTR without competing against the drug but rather stabilizing its binding. The effective capacity of NAM to bind F508del-CFTR in a lumacaftor-uncompetitive manner was then validated experimentally by surface plasmon resonance analysis. Finally, the capacity of NAM to synergize with lumacaftor increasing its CFTR-rescuing activity was demonstrated in cell-based assays. This study suggests the possible identification of natural small molecules devoid of side effects and endowed with the capacity to synergize with drugs currently employed for the treatment of cystic fibrosis, which hopefully will increase the therapeutic efficacy with lower doses.

## 1. Introduction

Cystic fibrosis is the most common lethal monogenic disorder in Caucasians. It is caused by more than 2000 mutations in the cystic fibrosis transmembrane conductance regulator (CFTR) [1]. CFTR is an ion chloride channel located in the plasma membrane of epithelial cells. Its loss of function has an impact on various organs, including those of the respiratory system. Cystic fibrosis patients, due to the disruption of the extracellular water-salt balance, produce thick mucus that is not efficiently cleared. This accumulation causes an impairment of the innate defense against bacteria [2] and, in turn, bronchial and pulmonary infections and chronic inflammation. Pulmonary infections lead to a progressive loss of respiratory function that may require life-saving intervention, such as a lung transplant [3,4].

CFTR is composed of five domains: two nucleotide-binding domains (NBD1 and NBD2), two transmembrane domains, and one regulatory domain [5]. The mutations of CFTR are classified into six groups, according to their effect on the protein function [1,6]. The most common one is a group II mutation, which affects protein maturation. It is the deletion of phenylalanine 508 (F508del) and occurs in 70% of cystic fibrosis patients. According to the literature, over 2100 CFTR mutations have been described (Cystic Fibrosis Mutation Database, https://www.genet.sickkids.on.ca/, accessed on 27 July 2022). Most of them, however, are extremely rare compared to F508del. All together they respond for the remaining 30%, with only five mutations reaching an allele frequency above 1% and 20 above 0.3%. F508 is in the NBD1 domain, and its deletion leads to an inappropriate folding and structural instability of the protein. Thus, F508del-CFTR remains trapped in the endoplasmic reticulum and degraded by the ubiquitin/proteasome system.

Despite the success of several potentiators/correctors drug combinations that are currently on the market (see below), current cystic fibrosis therapies still need to include symptomatic treatments (e.g., aggressive antibiotic strategies, osmotic agents, and pancreatic enzyme products) which have significantly enhanced the mean survival age of patients [7,8]. Nevertheless, the burden of cystic fibrosis care remains very high, requiring both the development of both novel symptomatic treatments and potentiators/correctors drug combinations.

Modulators are drugs that control CFTR function by three distinct mechanisms: correctors, potentiators, and amplifiers, which restore the trafficking, repair the gating activity, and stabilize the mRNA of the mutated CFTR, respectively [9,10]. Over the years, thousands of these synthetic drugs have been evaluated, leading to the approval of three correctors (lumacaftor/VX809, tezacaftor/VX661, and elezacaftor/VX445) and one potentiator (ivacaftor/VX770), currently administered to cystic fibrosis patients in four different preparations: Kalydeco (VX770), Orkambi (VX809/VX770), Symdeko (VX661/VX770), and Trikafta (VX445/VX661/VX770) [11]. At a molecular level, the rationale for the use of these drugs in combination is based on the fact that they exert their actions by binding the mutated CFTR in different positions, synergizing and increasing the functional rescue of the protein. Two studies have identified the potential binding sites of the lumacaftor: one has used cryo-electron microscopy to identify the binding site in the membrane-spanning domain (MSD1) and reports the drug as able to induce the stabilization of MSD1, making it less sensitive to the ER degradation and restoring the tertiary structure of misfolded CFTR [12]. The second study, based on molecular docking and molecular dynamics, locates the lumacaftor in the first nucleotide-binding domain (NBD1), and reports the drug as able to induce the stabilization of the NBD1:ICL4 (IntraCellular Loop 4) interaction and the stabilization of the already-folded CFTR in its native conformation [12,13]. Similarly, a new low molecular weight (MW) compound (c407) has been recently described to rescue F508del-CFTR activity by binding the protein in a different site from that of lumacaftor, hence exerting a synergic effect when used in combination [14]. Accordingly, phenylsulfonamide-pyrrolopyridine acts synergistically with VX770 to increase the truncated W1282X-CFTR function [15]. Besides the benefits already brought to cystic fibrosis patients by CFTR modulators approved so far, novel drugs are eagerly awaited to design novel combinations that could further increase the efficacy of cystic fibrosis treatment.

Since 2012, industry and academia have largely used drug repositioning to accelerate drug development for rare genetic diseases [16,17]. To date, hundreds of drugs have been repurposed, with a parallel progressive decrease of the number of new molecules globally approved [18]. In the same period, the support offered by bioinformatics, and the availability of powerful computational resources (such as high computing platforms for big data management and analysis), has speeded up in silico drug repositioning [19] over biological, experimental, mixed and knowledge-based approaches [20,21,22].

Drug repositioning has already been used for cystic fibrosis [23,24]. In line with this, we have previously carried out a careful investigation around the lumacaftor binding region of F508del-CFTR [25]. The region identified within this study was in full agreement with the literature [13] (and literature cited therein). On the sub-regions surrounding the lumacaftor binding site [25] endowed with a high druggable probability score, we have performed a pilot computational drug repositioning analysis to identify new compounds with CFTR-rescuing potential. Interestingly, the natural compound rutin was identified as a hit and its metabolite quercetin also proved to bind the mutated protein, partially overlapping to the lumacaftor interaction region of F508del-CFTR [25].

Since many other nutraceuticals have demonstrated to alleviate cystic fibrosis symptoms by different mechanisms of action [26,27,28,29], we have attempted a computational drug repositioning analysis to identify small compounds able to accommodate in the large lumacaftor interaction region of F508del-CFTR in such a way to synergize with the drug. The drug repositioning analysis was followed by biochemical and biological experimental validation assays.

## 2. Results

### 2.1. Computational Drug Repositioning Pipeline to Identify Small Molecules Able to Interact with the Lumacaftor-Binding Region of F508del-CFTR

**Figure 1:**
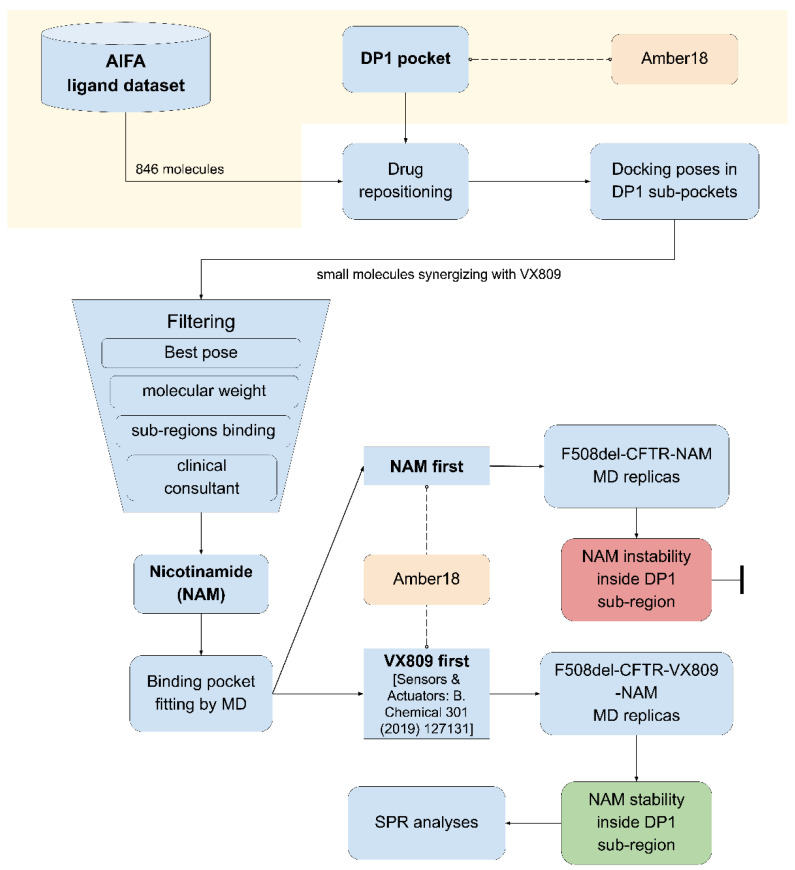
Schematic representation of the drug repositioning pipeline adopted in this work. Our previously published pipeline [25] was implemented to adapt it for the searching of small molecules able to combine with lumacaftor inside the DP1.

A putative binding site for lumacaftor located between NBD1 and ICL4 has already been identified [30]. The prediction, supported by experimental data [31,32], suggested the importance of a binding pocket we named DP1. Focused on this pocket, a first drug repositioning campaign was performed based on the Italian Drug Agency database (AIFA, Agenzia Italiana del FArmaco) [25]. The binding mode of the 11 repositioned drugs so far identified (all with a high molecular weight), highlighted that DP1 is a very large pocket that is not fully occupied by lumacaftor alone, or by one of the selected compounds. This observation prompted us to explore the several DP1 sub-pockets surrounding the lumacaftor binding region, searching for the most druggable ones [32], in the meantime scouting small molecules able to fill the druggable DP1 pocket and synergize with lumacaftor. To this aim, an ad hoc drug repositioning pipeline was updated (Figure 1) based on the previously developed one [25].

The drug repositioning results were evaluated based on 18 representative DP1 conformational pockets, obtained from molecular dynamics analysis of the F508del-CFTR model already published by us [30]. Among the 846 considered drugs, three typologies of binding sites can be defined, depending on residues involved in the binding inside DP1 [25]: the most populated group, corresponding to those drugs able to bridge NBD1 and NBD2, binding also to residues of the ICL4 domain. Among these, VX809; drugs that mostly occupy DP1 taking close interactions with residues in NBD1 and ICL4; drugs displaying interactions only with the apical region of NBD1.

The superimposition of one ligand for each group to VX809 highlighted three different space sub-regions around the aromatic acid portion of the template which are not occupied by VX809 itself but could be possibly occupied by other drugs. Following this premise, the docking results were filtered based on their binding into the druggable DP1 sub-regions, and ranked based on binding energy value and MW. After this step 1462 poses were obtained, corresponding to 517 molecules.

### 2.2. Clinical Assessment and Selection of the Repositioned Small Compounds

The first five ranked compounds (Table 1), being not part of the coded therapies for the treatment of cystic fibrosis patients, have been evaluated by the clinical consultant on the basis of the AIFA and EMA documents [www.farmaci.agenziafarmaco.gov.it (accessed on 27 July 2022) and www.ema.europa.eu (accessed on 27 July 2022) and references therein] to dischfarge those with side effects contraindicated for cystic fibrosis patients. Also, to highlight whether they could have any favorable effect in the treatment of cystic fibrosis, research on PubMed was performed [cystic fibrosis AND compound name]. As detailed below, three hits were excluded from further evaluation due to the presence of possible serious adverse effects and the consequent contraindication to use in cystic fibrosis in the absence of their specific therapeutic indications.

**Sodium oxybate** (also called gamma-hydroxybutyrate sodium) is a drug with a depressant action on the central nervous system used in adults and children older than 7 years of age to prevent cataplexy (sudden and short-time muscle weakness episodes) and narcolepsy (sudden uncontrollable urge to sleep during daily activities) [33]. It is also used in alcohol withdrawal syndrome and for the maintenance of abstinence in alcohol dependence [34]. In addition, sodium oxybate has a muscle relaxant effect and causes partial retrograde amnesia. Common adverse effects are enuresis and sleepwalking, while less common are lack of appetite, suicidal thoughts, trouble sleeping, unusual weight gain or loss, and changes in behaviour, creating a contraindication for cystic fibrosis patients [www.farmaci.agenziafarmaco.gov.it (accessed on 27 July 2022) and www.ema.europa.eu (accessed on 27 July 2022) and references therein].

**Methimazole** blocks T3 and T4 synthesis from the thyroid gland, and is useful in treating pathological conditions related to the thyroid, especially hyperthyroidism [35]. However, it can have particularly serious side effects, such as agranulocytosis, leukopenia, thrombocytopenia, and aplastic anemia. It is also hepatotoxic, causing hypoprothrombinemia and bleeding and thus requires the constant monitoring of liver function. Case reports of acute pancreatitis in patients treated with this drug have been noted, making it contraindicated for cystic fibrosis patients [www.farmaci.agenziafarmaco.gov.it (accessed on 27 July 2022) and www.ema.europa.eu and references therein (accessed on 27 July 2022)].

**Glycine betaine** is a methyl group donor that functions in the normal metabolic cycle of methionine and reduces homocystinuria in patients with inborn errors of methionine metabolism, as in the case of Homocystinuria, where treatment aims at reducing homocysteine accumulation, as in an alternative pathway that aims to eliminate the toxic substrate (i.e., pharmacologic treatment with betaine which re-methylates homocysteine to methionine) [36]. With regard to safety, betaine appears to be well tolerated. Its adverse effects, although not serious, are mainly related to the gastrointestinal system that, importantly, could already be compromised in cystic fibrosis patients. Furthermore, no data has been found on its possible use in cystic fibrosis [www.farmaci.agenziafarmaco.gov.it (accessed on 27 July 2022) and www.ema.europa.eu (accessed on 27 July 2022) and references therein].

Two compounds were considered usable in patients with cystic fibrosis, namely cysteamine and NAM, whose structural and functional features and known therapeutic properties and possible use in cystic fibrosis are detailed here below.

**Cysteamine** is derived from coenzyme A degradation. It is an AIFA and FDA-approved drug originally prescribed for nephropathic cystinosis, with known adverse effects limited to myopathy, diabetes, and hypothyroidism [37]. Over the years, it has also been used for neurodegenerative disorders and non-alcoholic fatty liver disease [38]. Interestingly, cysteamine has already been the subject of intense studies in the field of cystic fibrosis, with 34 publications retrieved from the research on PubMed. Relevantly, oral cysteamine is absorbed and enters the bronchial secretions in patients with cystic fibrosis, and its activity is not sensitive to high ionic concentrations characteristic of the cystic fibrosis lung [39]. Accordingly, its therapeutic benefit has been proved even in patients [40,41] where, although adverse reactions are common, it appears to be safe and well-tolerated [42]. Therapeutic benefits observed in cystic fibrosis patients treated with cysteamine are due to multiple effects exerted by the drug: it is endowed with autophagy-stimulatory proteostasis regulatory and anti-oxidant properties, and is able to exert a reduction of lung inflammation and to restore bacterial internalization and clearance in cystic fibrosis impaired macrophages [43,44]. Moreover, cysteamine exerts an antibacterial/anti-biofilm activity against cystic fibrosis relevant pathogens [45]. Interestingly, cysteamine has been originally proposed to rescue and stabilize F508del-CFTR at the plasma membrane [46], but following dedicated experiments with appropriate cell-based assays, it demonstrated that it does not exert any F508del-CFTR-rescuing activity when administered both alone or in combination with lumacaftor [47,48].

**NAM** (or niacinamide) is a water-soluble vitamin, the amide of vitamin B3 also known as Vitamin PP (Pellagra Preventing). It is a constituent of two coenzymes [nicotinamide adenine dinucleotide (NAD) and nicotinamide adenine dinucleotide phosphate (NADP)] which act as hydrogen and electron carriers through oxidation and reversible reductions and play a vital role in cellular metabolism. Therefore, NAM is an important precursor of NADH and NADPH. There are no special warnings and precautions for its use, and no significant pharmacokinetic or pharmacodynamic interactions with other medicines emerged from the analysis of the scientific literature. Only potential side effects have been suggested for its excessive exposure [49]. Interestingly, it has been suggested to exert a protective effect on acute lung damage caused by ischemia, endotoxin, or oxidative stress [50]. These features make it usable in patients with cystic fibrosis. On the basis of the lack of F508del-CFTR-rescuing activity already demonstrated for cysteamine [47,48] and of the novelty of a possible use of the natural compound NAM for the rescue of the CFTR function, we decided to consider the latter compound in this study.

### 2.3. Pocket Fitting Evaluation for Lumacaftor and NAM: Which Molecule Binds First?

**Figure 2:**
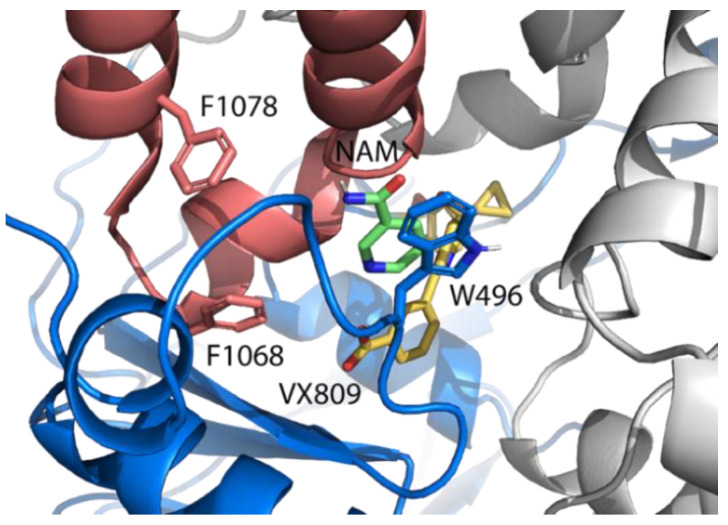
Docking pose of NAM in combination with the F508del-CFTR-lumacaftor structure. In blue is the NBD1 domain and in red is the ICL4.

NAM was selected as a probable hit. As shown in Figure 2, the docking pose obtained from drug repositioning located the molecule in a sub-region of DP1, in the interface between NBD1 and ICL4. It is close to the hydrophobic cluster between F508, W496, F1068, and, partially, with F1074, which is disrupted by the F508 deletion [51]. This made NAM very interesting for the F508del-CFTR rescue activity because it could be able to perform a recovery by itself or in combination with lumacaftor.

To investigate this second possibility, NAM was evaluated by MDs in combination with lumacaftor considering the protein dynamic behavior at the DP1. With this aim, we tried to evaluate the fitting determined by the binding of the first ligand, which induces modifications on DP1, so as to allow the binding of the second one. Two different MDs experiments of three replicas were set up. The first was to evaluate the fitting of lumacaftor [30] or NAM in the apo F508del-CFTR, and the latter to evaluate the stability of NAM in complex with the representative conformation of F508del-CFTR-lumacaftor and, vice versa, the stability of lumacaftor in complex with F508del-CFTR after NAM binding. The analyses of the three 50 ns MDs replicas of the F508del-CFTR-NAM complex highlighted that the NAM pose was unstable since the molecule was localized in three different binding poses (see Supplementary Figure S1). As a consequence, we hypothesized that it was lumacaftor the one to produce a pocket fitting for the binding of NAM into DP1. Thus, MDs of the complex including both molecules were performed.

**Figure 3:**
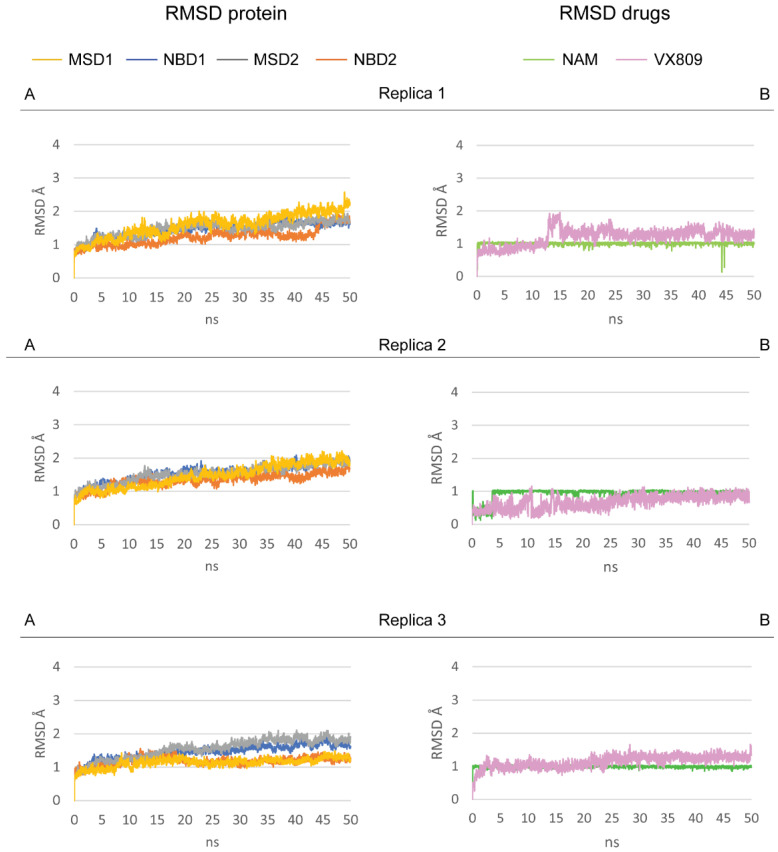
RMSD analysis of F508del-CFTR four domains (**A**), and NAM and lumacaftor (**B**) for the three replicas.

The NAM from drug repositioning was docked in the representative F508del-CFTR-lumacaftor structure determined in our previous studies [30], and the complex stability was evaluated by three 50 ns MDs replicas that were carried out. The analyses of the complex demonstrated an overall good binding pose of NAM and lumacaftor inside the DP1. RMSD analyses of F508del-CFTR, lumacaftor, and NAM showed an overall stability of the system (Figure 3).

Moreover, the two drugs showed an RMSD value < 2 Å in all three replicas from the starting conformation as showed in Figure 3. This indicates a good ligand stability [52].

**Figure 4:**
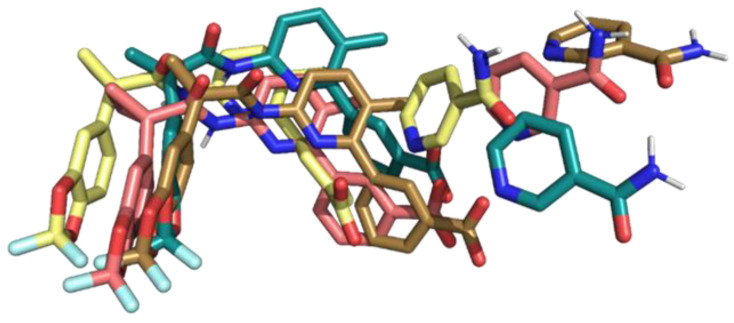
Binding pose of NAM and lumacaftor in the representative conformations of the three replicas. Conformation 1 of replica 1 in teal, conformation 1 of replica 2 in coral, conformation 2 of replica 2 in brown, and conformation 1 of replica 3 in yellow.

Then, to better study the binding pose of the drugs, a hierarchical cluster analysis for each replica was performed. A total of four representative conformations of the F508del-CFTR-lumacaftor-NAM complex were obtained: one for replica 1 representative of 76%, two for replica 2 representative of 47% and 33%, respectively, and one for replica 3 representative of 70% of clustered conformations. Conformation analysis indicated that lumacaftor maintains a similar binding pose in all replicas, while slight differences were found for NAM (Figure 4).

Only in replica 3 did lumacaftor show a different orientation of its benzoic acid moiety, due to a peculiar positioning of NAM inside the DP1 sub-region. H-bond analysis along MDs of lumacaftor showed differences in its interaction in comparison to its complex with the apo F508del-CFTR [30]. In the combined analyses, due to the presence of NAM, lumacaftor only interacted with W1063 (83.7%, 79.5%, and 84.1% for replica 1, 2, and 3, respectively). The variation of lumacaftor hydrophobic interactions with the representative conformation complex was evaluated by PLIP [53] and along the replicas’ trajectories.

To calculate these values, we analyzed the distances between the hydrophobic portion of the protein residues and the interacting hydrophobic portion of lumacaftor among the trajectories, with 6 Å as the cut-off value for the distance between the center of mass of the hydrophobic atoms. The analysis highlighted that lumacaftor interacted with F494, K1060, and W1063, in agreement with our previous studies [30]. Moreover, lumacaftor created stable hydrophobic interactions with L172, D173, and I177 of NBD1, and D1341 of NBD2 (Table 2).

**Figure 5:**
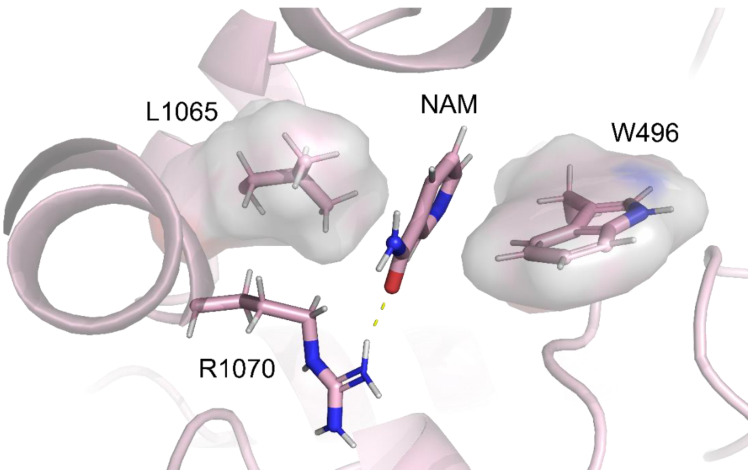
NAM binding pose in the F508del-CFTR-lumacaftor-NAM complex. The H-bond is shown in yellow dotted lines. Residues involved in hydrophobic interactions with NAM are depicted with their hydrophobic surfaces.

Concerning the binding pose of NAM inside the DP1 sub-region (Figure 5), we analyzed its molecular interactions with the protein. H-bond analysis highlighted a strong interaction between NAM and R1070 of ICL4 (66,4%, 77,4%, and 83.6% for replica 1, 2, and 3, respectively).

A hydrophobic interactions analysis, performed as described above for lumacaftor, indicated the interaction of NAM with W496 of NBD1 and L1065 of ICL4 (Table 2). Visual inspection of NAM binding poses along the three replicas showed that the molecule is anchored inside the DP1 sub-pocket, with a single H-bond with an R1070 side chain. Comparing NAM representative conformations obtained from replicas, we observed that R1070 residue, with its side chain, drags NAM, anchored by an H-bond, in a spatial restraint around 6.13 Å. Despite this defined spatial movement determined by R1070 side chain, the NAM hydrophobic network is preserved thanks to the NAM pyridinic ring, which is steadily interacting with residues L1065 and W496. These results highlighted the ability of NAM to occupy a very specific DP1 sub-region inside the F508del-CFTR, exploiting an H-bonding and several hydrophobic interactions (when lumacaftor is bound).

F508del mutation involves the deletion of a phenylalanine in NBD1 which contributes to generate molecular contacts at the ICL4/NBD1 interface. The elimination of F508 causes a disruption of a hydrophobic cluster formed by residues F508, W496, F1068 and F1074, located at the interface between NBD1 and ICL4. The hydrophobic interactions between the NAM’s pyridine moiety and residues W496 and L1065, both located in the ICL4 loop, might help in restoring this compromised interaction between ICL4 and NBD1. According to our calculations, NAM is able to restate that interaction between W496 and F508, which is lost after mutation, and in addition is able to interact with L1065, a residue recently defined as able to revert F508del mutation effects on CFTR [54].

### 2.4. Biochemical Validation: Binding Assay of NAM to F508del-CFTR by Surface Plasmon Resonance

**Figure 6:**
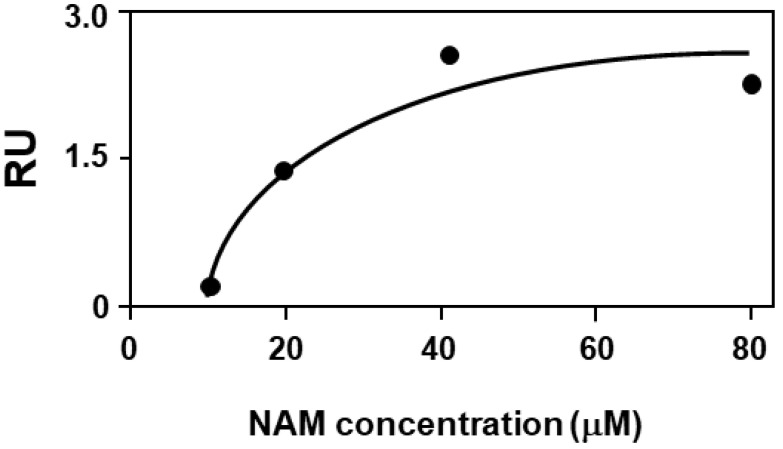
Steady-state analysis of NAM injected onto the F508del-CFTR-containing biosensor. The results shown are representative of other seven analyses that gave similar results.

In our previous works, we have successfully exploited surface plasmon resonance to validate virtual binding predicted by computational studies [25,30,55]. We then decided to use this technique to demonstrate the effective capacity of NAM to bind to F508del-CFTR. A biosensor containing F508del-CFTR immobilized in membrane-like lipid vesicles that resembles the F508del-CFTR environment in vivo and that has already allowed the successful validation of computational predictions in the field of cystic fibrosis [30,56,57] was used. As shown in Figure 6, when injected at increasing concentrations onto the F508del-CFTR-containing a biosensor, NAM binds the mutated protein in a dose-dependent and saturating manner with a Kd equal to 2.5 ± 1.3 µΜ (value + SEM obtained from seven independent analyses). As described in the Section 4, the use of a control surface devoid of the target protein for blank subtraction proves the specificity of the observed binding, further sustained by the saturation binding, further sustained by the saturation binding reached at higher concentration of NAM.

Interestingly, in the same experimental conditions, lumacaftor binds F508del-CFTR with a significantly higher Kd (20–30 times) than NAM [30,57].

### 2.5. Identification of the Binding Pocket of NAM

**Figure 7:**
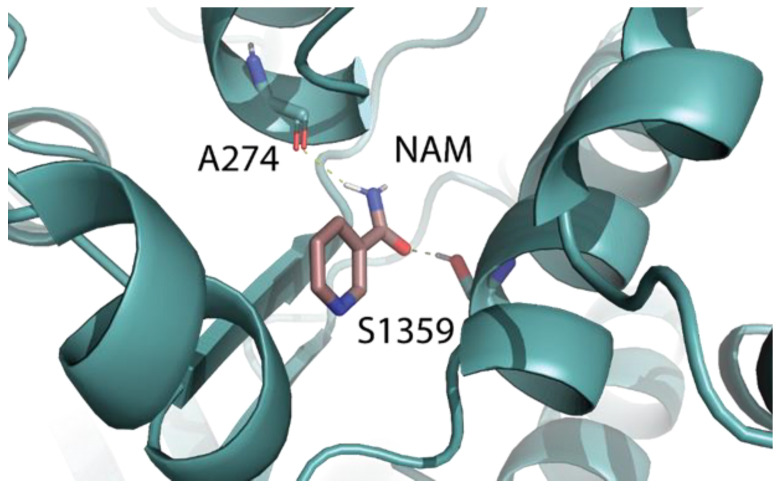
Binding pose of NAM in the ICL2:NBD2 interface when it binds to the apo F508del-CFTR. H-bond in yellow.

Surface plasmon resonance demonstrated the NAM/F508del-CFTR interaction without providing the location of the binding site. Relevantly, molecular docking is instrumental in the prediction of binding site(s) [58], prompting us to use it for our aims. Due to the instability of NAM alone inside the DP1 sub-region, we computationally evaluated the binding of NAM outside the DP1. NAM was docked against the apo F508del-CFTR pocket library [30]. The best docking pose (−6.1 Kcal/mol) located in the ICL2:NBD2 interface was selected for further analysis. The complex between the docking pose of NAM and the apo F508del-CFTR was evaluated by 50 ns MDs. The RMSD analysis showed the stability of NAM (Figure 3). As shown in Figure 7, the binding of NAM occurs through an H-bond interaction with A274 (ICL2) and S1359 (NBD2) for 72.6% and 99.5% of lifetime along the trajectory, respectively. No hydrophobic interactions were found.

### 2.6. Effect of NAM-Lumacaftor Co-Treatment on Mutant CFTR Rescue

**Figure 8:**
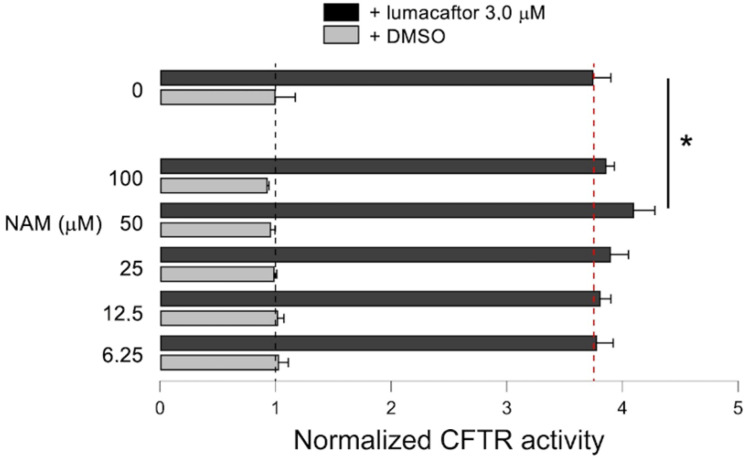
Effect of NAM-lumacaftor co-treatment on mutant F508del-CFTR rescue. The bar graphs show F508del-CFTR activity in CFBE41o-cells stably expressing the HS-YFP. CFTR activity was determined as a function of the YFP quenching rate following iodide influx in cells treated for 24 h with DMSO in the absence (vehicle) or in the presence of lumacaftor (3.0 µM) as a single agent or combined with the indicated concentrations of NAM. * *p* < 0.05.

We tested the effect of NAM alone and in co-treatment with lumacaftor on F508del-CFTR activity by using the Halide-Sensitive Yellow Fluorescent Protein (HS-YFP) assay. The assay was performed on immortalized bronchial CFBE41o- cells stably expressing the HS-YFP and the mutant F508del-CFTR. Cells were treated for 24 h with lumacaftor (3 µM) in the absence or in the presence of increasing concentrations of NAM (from 6.25 to 100 µM). The compounds were then removed and the cells were assayed after stimulating CFTR activity with forskolin (20 µM) and VX-770 (1 µM). Incubation with NAM alone does not increase CFTR activity as compared to treatment with vehicle alone (DMSO), while lumacaftor alone (3 µM) caused a 3.7-fold improvement in CFTR-mediated transport. However, when cells were co-treated with lumacaftor and increasing concentrations of NAM, a bell-shaped dose response was observed, with a modest but significant increase in the rate of YFP quenching observed with NAM at 50 μM that suggests an augmented CFTR rescue and expression at the plasma membrane (Figure 8). Further analysis is needed to investigate the biological significance of the improved rescue in more relevant cystic fibrosis cell models.

## 3. Discussion

Cystic fibrosis treatment still relies on antimicrobial agents, making patients vulnerable to the development of drug resistances. A great effort has already been undertaken to identify alternative pharmacological targets with important results achieved in the development of efficacious combinations of CFTR-rescuing drugs. However, these combinations can be used only for patients older than age 12. Also, although they can be administered to nearly 90% of cystic fibrosis patients, a 10% of cystic fibrosis patients remain without etiological therapies [59]. Finally, they will not be available worldwide due to their cost.

In this scenario, the identification of agents endowed with a dual-target activity (i.e., antibacterial and antioxidant or antioxidant and CFTR-rescuing activities combined in one molecule) may lead to increased therapeutic benefits for cystic fibrosis patients [60].

On the other hand, the dietary implementation of nutraceuticals in cystic fibrosis treatment has been considered [61,62,63]. Accordingly, CFF USA Registry reported in 2020 that about 40% of CF patients required oral nutrition supplements, as minerals (sodium chloride, magnesium, zinc), fat-soluble vitamins (A, D, E, K), fatty acids, and probiotics. Probiotics can intervene on CF intestinal dysbiosis, reducing its negative effects (i.e., severe intestinal inflammation). Moreover, they may have a positive effect on glucose metabolism [64,65]. Fatty acids, vitamins (A, D, E, K and B12) [26,27,28,29,66,67], flaxseed [68], caffeic acid phenethyl ester [69], furocoumarins [70], curcumin, resveratrol, genistein [63,71,72,73] and rutin/quercetin [74,75] are endowed with antioxidant potential that may compensate the oxidative stress caused by the increased production of ROS during chronic pulmonary infections. They can reduce airway inflammation and/or increase survival in animal models and/or in cystic fibrosis patients. Interestingly, beside their anti-oxidant potential, some of these natural compounds are also endowed with CFTR-rescuing activity: genistein and resveratrol act as an F508del-CFTR potentiator [76,77] and furocoumarins that improve the chloride channel transport of the mutant CFTR protein [70]. Rutin and its metabolite quercetin are endowed with a CFTR-rescuing activity [78] that could depend, at least in part, on their capacity to bind directly to mutated CFTR [13].

Finally, it is interesting to note how some natural compounds (including genistein, digitoxigenin, curcumin, resveratrol, and latonduine) show additive/synergic effects when administered in combination with already approved cystic fibrosis drugs [79,80,81,82,83].

Taken together, these observations call for the systematic search of nutraceuticals with CFTR-rescuing potential for treatment in cystic fibrosis, an aim that may be achieved by drug repositioning that has already been used both for cystic fibrosis [23,24] and for screening natural products in various pathological settings [84]. Based on this, we have set up a virtual drug repositioning pipeline specifically aimed at screening small molecules endowed with F508del-CFTR-binding and rescuing capacity, from which we selected NAM for further study. NAM is the main precursor of NAD+ that, in turn, is an essential co-enzyme of redox reactions for adenosine triphosphate (ATP) production and ATP-dependent metabolic processes critical in maintaining cellular energy. NAD+ and its precursor NAM are therefore essential for metabolically active tissues such as epithelia. Relevant to cystic fibrosis, NAM exerts a protective effect on acute lung damage caused by ischemia, endotoxin, or oxidative stress [50], and reduces the levels of pro-inflammatory cytokines, inducible nitric oxide synthase, and other cellular and biochemical inflammation markers [85]. Interestingly, the NAM intracellular pyridine nucleotides derivatives have been demonstrated to regulate CFTR-mediated CAMP-dependent Cl- conductance [86], strengthening the hypothesis that NAM may positively affect CFTR activity, pointing to NAM as a putative multitarget compound able to act on different pathological aspects of the cystic fibrosis disease (i.e., reducing oxidation, inflammation and CFTR conductance).

Here, by a dedicated pipeline, a drug repositioning study supported by molecular docking and dynamics predicted NAM to bind to F508del-CFTR into the lumacaftor binding pocked DP1 located in the NBD1 domain and partially interfaced with the ICL4 domain, a dynamic interface crucial for CFTR gating [54]. Interestingly, NAM created a hydrophobic interaction with W496 and L1065. W496 is a residue which is part of the hydrophobic pattern altered by the deletion of F508. In wild-type CFTR, W496 forms a hydrophobic cluster with F508 itself, F1068, and, partially, with F1074 [51]. Moreover, in full agreement with the recently published paper by Prins et al. [54], NAM also interacts with L1065, a revertant residue able to partially rescue F508 deletion. Furthermore, the binding of NAM to R1070 could have biological importance. In literature it is reported how R1070W mutation acts by restoring interactions at the ICL4/NBD1 interface and thus reinstates the protein functionality compromised by F508 deletion [87]. In the same way, NAM bound to R1070 could partially mimic F508, helping the F508del-CFTR rescue.

In conclusion, the binding of NAM in the NBD1:ICL4 interface and its interaction with L1065, R1070 and W496 allow us to hypothesize a synergic effect between lumacaftor and NAM in F508del-CFTR rescue.

Surface plasmon resonance binding analysis confirmed experimentally that NAM effectively binds to F508del-CFTR with an affinity that is even higher than that of lumacaftor but cell-based assays demonstrated it does not rescue CFTR function when assayed alone in those same experimental conditions in which lumacaftor results effective. Instead, a preliminary evaluation of the effects of NAM/lumacaftor co-treatment on immortalized bronchial cells demonstrated a modest improvement in mutant CFTR rescue. The dissociation between CFTR-binding and rescuing activity of NAM could be caused by the fact that, when alone, NAM binds to the ICL2:NBD2 interface which, to date, is not known to be implicated in the F508del-CFTR rescue. At variance, when NAM is administered in combination with lumacaftor, the binding of the latter could contribute to the subsequent binding of NAM to the DP1 sub-region at the NBD1:ICL4 interface, synergizing with lumacaftor for the F508del-CFTR rescue. Furthermore, the fact that NAM is a very small molecule (MW equal to 122 Da) could account for its capacity to be accommodated in the DP1 sub-region without competing with lumacaftor, but rather synergizing in correcting the F508del-CFTR folding defect.

When administered in combination with lumacaftor, NAM increases the rescuing activity of lumacaftor in a bell-shaped way. Amazingly, a similar dose-response is also displayed by digitoxigenin when administered in combination with lumacaftor, a behavior that has been tentatively explained with a toxic effect exerted by the higher doses of digitoxigenin [83]. However, NAM has been demonstrated to decrease cell viability in culture only at 5 mM [88], a dose that is 50 times higher than those used in our experiments. On the other hand, genistein has also been demonstrated to affect CFTR activity in a bell-shaped way, possibly due to its interaction at two binding sites of the mutated protein: a high affinity site that decreases the closing rate and a low affinity site that reduces the opening rate [89]. It is thus tentative to hypothesize that, at lower doses, NAM adopts the high affinity binding mode here described that leads to the synergism with lumacaftor in rescuing CFTR activity while, at higher doses, low affinity aspecific binding(s) could occur that counteract the rescuing effect. Further investigation is warranted, although they are beyond the scope of the present work.

The combination of drugs is currently an almost mandatory approach to increasing the therapeutic benefits of the treatment of cystic fibrosis [11]. Among drug combinations already approved for clinical use, Orkambi and Symdeko contain correctors and potentiators that act by distinct mechanisms of action in turn mediated by a direct binding of the drugs to different regions of the mutated protein. [30,90]. Furthermore, the combination of three modulators (Trikafta) show a synergic effect that is achieved, once again, by the binding of the drugs to different CFTR regions [91]. Finally, the CFTR-rescuing activity of lumacaftor is increased when it is used in combination with digitoxigenin [83] and with C407, another corrector that binds the mutated CFTR in the same region here identified for NAM [14]. Interestingly, two of the three residues interacting with NAM (W496 of NBD1 and R1070 of ICL4) were found to also interact with C407 [14].

In addition to these combinations, our data suggest an alternative approach consisting of structural modifications of known CFTR-rescuing molecules (such as lumacaftor) including the moiety corresponding to a small molecule (such as NAM) that, even if devoid of intrinsic CFTR-rescuing activity, could stabilize the whole binding to the mutated protein, increasing the global CFTR-rescuing effect.

## 4. Materials and Methods

### 4.1. Drug Repositioning Pipeline

The druggable pocket of lumacaftor (DP1), obtained from the F508del-CFTR-lumacaftor complex [30], was already used by us to perform drug repositioning by docking analysis (see Section 4.3) using the AIFA ligand dataset (see Section 4.2). Here, a new drug repositioning procedure was applied scouting small molecules able to induce DP1 fitting upon binding in combination with lumacaftor in the same pocket by further re-examining the previous drug repositioning results. Results were evaluated as follows: the best pose of each drug was selected and further filtered based on its molecular weight (<500 Da), binding in DP1 sub-regions, and pharmacological effects. Ultimately, nicotinamide (NAM) was found as a hit. Subsequently, the F508del-CFTR-lumacaftor-NAM complex stability was evaluated by 50 ns of molecular dynamic simulations (MDs) using the Amber18 package (see Section 4.4). Two series of MDs were carried out: firstly, three MDs replicas were performed using the complex between the selected docking pose of NAM inside the DP1 sub-region of the apo F508del-CFTR conformation to evaluate the binding pose of NAM inside the DP1 sub-region of the apo F508del-CFTR. Relevant to this point, data concerning the binding of lumacaftor to the apo F508del-CFTR has already been published [30]. Then, three MDs replicas of the selected NAM docking pose in complex with the representative conformation of the F508del-CFTR-lumacaftor complex [30] were performed to assess the ability of the two drugs to combine in the DP1. MDs were eventually followed by surface plasmon resonance analyses (see Section 4.7) to experimentally validate the computational findings.

### 4.2. AIFA Ligand Dataset

The AIFA dataset, already described [25], was used to perform drug repositioning. It is a manually curated dataset for computational studies comprehensive of 846 approved drugs. All 3D conformations of each drug, keeping into account one or more chiral center, were drawn at physiological pH 7.4.

### 4.3. Molecular Docking Analysis

The eighteen F508del-CFTR conformations (17 for the apo F508del-CFTR and 1 for F508del-CFTR in complex with lumacaftor) collected from previous MDs studies [30] were used to perform drug repositioning on the DP1 [25] by Autodock Vina [92]. Hydrogens and Gasteiger charges [93] were added to F508del-CFTR conformations and pdbqt format files were generated as input for Autodock Vina. For each molecule in the 3D conformer of the AIFA dataset, Gasteiger charges were added, and the structures were converted into pdbqt format for use in docking calculations with Vina.

Semi-flexible docking was used to allow the ligands to sample various conformations during a semi-flexible docking stage, whereas the protein receptor was kept rigid. Default parameters were applied except for exhaustiveness, which was set to 24. The receptor-docking site was within the DP1 pocket, with the grid boxes centered on its geometric center. The docking results were filtered based on their binding into the druggable DP1 sub-regions, ranked based on binding energy value and MW and then analyzed.

### 4.4. Molecular Dynamic Simulations

MDs were performed using AMBER-18 [94] with the ff14SB force field for the protein [95] and lipid14 for the DOPC lipid bilayer [96]. The complexes were solvated with the TIP3P water model and neutralized by the addition of counter ions. Following the parameterization used for the simulations: (1) Thirteen minimizations for a total of 55,000 steps of steepest descent minimization and 55,000 steps of conjugate gradient, with decreasing restraints on the whole system and non-bonded cut-off set at 8 Å. The reaching of the system energy plateau was confirmed; (2) heating at constant volume (NVT), increasing the temperature from 0 K to 300 K as follows: 0–100 K in 50 ps then further 50 ps at constant temperature 100 K, using 1 kcal/mol restraint for protein (and lumacaftor when present) and 4 kcal/mol for NAM in both steps; 100–300 K in 75 ps, then further 25 ps at constant temperature 300 K, decreasing the restraint on NAM to 2 kcal/mol. The reaching of the desired temperature was confirmed. From the initial step of the heating, each independent simulation was carried out using different initial velocities; (3) 4 ns of equilibration at constant pressure (NPT) at 300 K. Protein and ligand(s) were either restrained with 1 kcal/mol; (4) 50 ns MDs production; or without any restraint. All of the trajectory analyses were carried out using AmberTools (cpptraj), evaluating the binding pose of the ligand and the stability of the protein.

### 4.5. Data Curation

The druggable pocket used in this study was obtained from the F508del-CFTR-lumacaftor complex published by us in [30], the AIFA ligand dataset used for the drug repositioning was created in house. Molecular docking and molecular dynamic simulations were performed with Autodock Vina [https://vina.scripps.edu/] and the Amber18 package (https://ambermd.org) available under license.

### 4.6. Reagents

His-tagged human intact F508del-CFTR protein was purified as described [97,98]. Lipids [synthetic phospholipid blend (Dioleoyl) DOPC:DOPS [(7:3 *w*/*w*)] were from Avanti Polar Lipids, Alabaster, AL. CHAPS and cholesteryl hemisuccinate (CHS) Tris salt was from Sigma-Aldrich, St Louis, MO, USA. Carboxy-methyl dextran CM5 sensor chip, anti-His antibody, 1-ethyl-3-(3-diaminopropyl)-carbodiimide hydro-chloride and N-hydroxysuccinimide (NHS) were from GE-Healthcare, Milwaukee, WI, USA. Lumacaftor was from Selleck Chemicals, Houston TX, USA. NAM was from TargetMol, Wellesley Hills, MA, USA.

### 4.7. Surface Plasmon Resonance

A BIAcore X-100 instrument (GE-Healthcare) was used. The biosensor containing F508del-CFTR in a membrane-like lipid environment was prepared as described [30]. A sensor chip coated with anti-His antibody alone was used for blank subtraction and hence the determination of a specific binding to F508del-CFTR to evaluate the affinity of its binding. NAM was injected over the sensor chip at increasing concentration in PBS, 0.05% surfactant P20 and 5% DMSO, pH 7.4 by adopting the single-cycle model [99]. Dissociation constant (Kd) values were calculated by steady-state analyses performed by fitting the proper form of Scatchard’s equation for the plot of the bound resonance units (RU) at equilibrium versus the compound concentration in solution by means of the BIAEVALUATION software embedded in the BIAcore X-100 instrument. Lumacaftor was used as positive control. For competition assays, Lumacaftor (75 µM) and increasing concentrations of NAM were injected onto the F508del-CFTR-biosensor and the effect of NAM on the binding of Lumacaftor to the immobilized protein was evaluated.

### 4.8. YFP-Based Assay for CFTR Activity

The microfluorimetric assay based on the HS-YFP that measures CFTR-mediated iodide/chloride transport was already described [100,101,102,103]. In brief, cells co-expressing HS-YFP and F508del-CFTR were incubated for 20 min in PBS containing forskolin (20 µM) and VX-770 (1 µM) to activate CFTR and then transferred to a microplate reader (FluoStar Galaxy or Fluostar Optima; BMG Labtech, Offenburg, Germany) equipped with YFP filters (Chroma Technology). HS-YFP fluorescence was registered for 14 s (2 s before and 12 s after the addition of an iodide-substituted PBS; Cl- replaced by I-; final I- concentration 100 mM). The iodide influx rate was determined by fitting the final 11 s of the data for each well (after normalization to the initial background-subtracted fluorescence) with an exponential function to extrapolate the initial slope (dF/dt).

## Figures and Tables

**Table 1:** Hits resulting from the drug repositioning, ranked by increasing molecular weight.

Molecule Name	Molecular Weight (g/mol)	Docking Score (Kcal/mol)	2D Structure
Cysteamine	77.15	−2.7	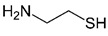
Sodium oxybate	104.17	−4.4	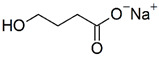
Methimazole	114.17	−3.6	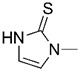
Glycine betaine	118.16	−3.9	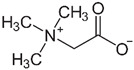
Nicotinamide	122.13	−5.4	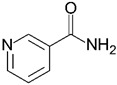

**Table 2:** Average distances of the three replicas between the hydrophobic portion of lumacaftor and NAM with the interacting F508del-CFTR hydrophobic portion residues.

**Lumacaftor**	L171 (ICL1)	L173 (ICL1)	I177 (ICL1)	F494 (NBD1)	K1060 (ICL4)	K1063 (ICL4)	D1341 (NBD2)
	5.05 Å	3.95 Å	4.92 Å	5.55 Å	4.45 Å	4.11 Å	5.14 Å
**NAM**	W496 (NBD1)	L1065 (ICL4)					
	5.31 Å	4.53 Å					

## Data Availability

Not applicable.

## Supplementary Materials

The following supporting information can be downloaded at: https://www.mdpi.com/article/10.3390/ijms232012274/s1.

## References

[1] Veit G., Avramescu R.G., Chiang A.N., Houck S.A., Cai Z., Peters K.W., Hong J.S., Pollard H.B., Guggino W.B., Balch W.E. (2016). From Cftr Biology toward Combinatorial Pharmacotherapy: Expanded Classification of Cystic Fibrosis Mutations. Mol. Biol. Cell.

[2] Rivas Caldas R., Boisrame S. (2015). Upper Aero-Digestive Contamination by Pseudomonas Aeruginosa and Implications in Cystic Fibrosis. J. Cyst. Fibros..

[3] Scott-Ward T.S., Amaral M.D. (2009). Deletion of Phe508 in the First Nucleotide-Binding Domain of the Cystic Fibrosis Transmembrane Conductance Regulator Increases Its Affinity for the Heat Shock Cognate 70 Chaperone. FEBS J..

[4] Amaral M.D., Farinha C.M. (2013). Rescuing Mutant Cftr: A Multi-Task Approach to a Better Outcome in Treating Cystic Fibrosis. Curr. Pharm. Des..

[5] Rogan M.P., DStoltz A., Hornick D.B. (2011). Cystic Fibrosis Transmembrane Conductance Regulator Intracellular Processing, Trafficking, and Opportunities for Mutation-Specific Treatment. Chest.

[6] Fanen P., Wohlhuter-Haddad A., Hinzpeter A. (2014). Genetics of Cystic Fibrosis: Cftr Mutation Classifications toward Genotype-Based Cf Therapies. Int. J. Biochem. Cell Biol..

[7] Elborn J.S. (2016). Cystic Fibrosis. Lancet.

[8] Bierlaagh M.C., Muilwijk D., Beekman J.M., van der Ent C.K. (2021). A New Era for People with Cystic Fibrosis. Eur. J. Pediatr..

[9] Dukovski D., Villella A., Bastos C., King R., Finley D., Kelly J.W., Morimoto R.I., Hartl F.U., Munoz B., Lee P.S. (2020). Amplifiers Co-Translationally Enhance Cftr Biosynthesis Via Pcbp1-Mediated Regulation of Cftr Mrna. J. Cyst. Fibros..

[10] Ponzano S., GNigrelli, Fregonese L., Eichler I., Bertozzi F., Bandiera T., Galietta L.J.V., Papaluca M. (2018). A European Regulatory Perspective on Cystic Fibrosis: Current Treatments, Trends in Drug Development and Translational Challenges for Cftr Modulators. Eur. Respir. Rev..

[11] Habib A.R., Kajbafzadeh M., Desai S., Yang C.L., Skolnik K., Quon B.S. (2019). A Systematic Review of the Clinical Efficacy and Safety of Cftr Modulators in Cystic Fibrosis. Sci. Rep..

[12] Fiedorczuk K., Chen J. (2022). Mechanism of Cftr Correction by Type I Folding Correctors. Cell.

[13] Baatallah N., Elbahnsi A., Mornon J.P., Chevalier B., Pranke I., Servel N., Zelli R., Decout J.L., Edelman A., Sermet-Gaudelus I. (2021). Pharmacological Chaperones Improve Intra-Domain Stability and Inter-Domain Assembly Via Distinct Binding Sites to Rescue Misfolded Cftr. Cell. Mol. Life Sci..

[14] Bitam S., Elbahnsi A., Creste G., Pranke I., Chevalier B., Berhal F., Hoffmann B., Servel N., Baatalah N., Tondelier D. (2021). New Insights into Structure and Function of Bis-Phosphinic Acid Derivatives and Implications for Cftr Modulation. Sci. Rep..

[15] Haggie P.M., Phuan P.W., Tan J.A., Xu H., Avramescu R.G., Perdomo D., Zlock L., Nielson D.W., Finkbeiner W.E., Lukacs G.L. (2017). Correctors and Potentiators Rescue Function of the Truncated W1282x-Cystic Fibrosis Transmembrane Regulator (Cftr) Translation Product. J. Biol. Chem..

[16] Gordon L.B., MKleinman E., Miller D.T., Neuberg D.S., Giobbie-Hurder A., Gerhard-Herman M., Smoot L.B., Gordon C.M., Cleveland R., Snyder B.D. (2012). Clinical Trial of a Farnesyltransferase Inhibitor in Children with Hutchinson-Gilford Progeria Syndrome. Proc. Natl. Acad. Sci. USA.

[17] Roessler H.I., Knoers N., van Haelst M.M., van Haaften G. (2021). Drug Repurposing for Rare Diseases. Trends Pharmacol. Sci..

[18] Polamreddy P., Gattu N. (2019). The Drug Repurposing Landscape from 2012 to 2017: Evolution, Challenges, and Possible Solutions. Drug Discov. Today.

[19] Farghali H., Canova N.K., Arora M. (2021). The Potential Applications of Artificial Intelligence in Drug Discovery and Development. Physiol. Res..

[20] Pillaiyar T., Meenakshisundaram S., Manickam M., Sankaranarayanan M. (2020). A Medicinal Chemistry Perspective of Drug Repositioning: Recent Advances and Challenges in Drug Discovery. Eur. J. Med. Chem..

[21] Park K. (2019). A Review of Computational Drug Repurposing. Transl. Clin. Pharmacol..

[22] Lara-Ramirez E.E., Lopez-Cedillo J.C., Nogueda-Torres B., Kashif M., Garcia-Perez C., Bocanegra-Garcia V., Agusti R., Uhrig M.L., Rivera G. (2017). An in Vitro and in Vivo Evaluation of New Potential Trans-Sialidase Inhibitors of Trypanosoma Cruzi Predicted by a Computational Drug Repositioning Method. Eur. J. Med. Chem..

[23] Newman S.P. (2018). Delivering Drugs to the Lungs: The History of Repurposing in the Treatment of Respiratory Diseases. Adv. Drug Deliv. Rev..

[24] Anderson S.D., Daviskas E., Brannan J.D., Chan H.K. (2018). Repurposing Excipients as Active Inhalation Agents: The Mannitol Story. Adv. Drug Deliv. Rev..

[25] Orro A., Uggeri M., Rusnati M., Urbinati C., Pedemonte N., Pesce E., Moscatelli M., Padoan R., Cichero E., Fossa P. (2021). In Silico Drug Repositioning on F508del-Cftr: A Proof-of-Concept Study on the Aifa Library. Eur. J. Med. Chem..

[26] Dodge J.A., Turck D. (2006). Cystic Fibrosis: Nutritional Consequences and Management. Best Pract. Res. Clin. Gastroenterol..

[27] Portal C., Gouyer V., Leonard R., Husson M.O., Gottrand F., Desseyn J.L. (2018). Long-Term Dietary (N-3) Polyunsaturated Fatty Acids Show Benefits to the Lungs of Cftr F508del Mice. PLoS ONE.

[28] Fangous M.S., Lazzouni I., Alexandre Y., Gouriou S., Boisrame S., Vallet S., le Bihan J., Ramel S., Hery-Arnaud G., le Berre R. (2018). Prevalence and Dynamics of Lactobacillus Sp. In the Lower Respiratory Tract of Patients with Cystic Fibrosis. Res. Microbiol..

[29] Sagel S.D., Khan U., Jain R., Graff G., Daines C.L., Dunitz J.M., Borowitz D., Orenstein D.M., Abdulhamid I., Noe J. (2018). Effects of an Antioxidant-Enriched Multivitamin in Cystic Fibrosis. A Randomized, Controlled, Multicenter Clinical Trial. Am. J. Respir. Crit. Care Med..

[30] D’Ursi P., Uggeri M., Urbinati C., Millo E., Paiardi G., Milanesi L., Ford R.C., Clews J., Meng X., Bergese P. (2019). Exploitation of a Novel Biosensor Based on the Full-Length Human F508de1-Cftr with Computational Studies, Biochemical and Biological Assays for the Characterization of a New Lumacaftor/Tezacaftor Analogue. Sens. Actuators B-Chem..

[31] Hudson R.P., Dawson J.E., Chong P.A., Yang Z., Millen L., Thomas P.J., Brouillette C.G., Forman-Kay J.D. (2017). Direct Binding of the Corrector Vx-809 to Human Cftr Nbd1: Evidence of an Allosteric Coupling between the Binding Site and the Nbd1:Cl4 Interface. Mol. Pharmacol..

[32] Okiyoneda T., Veit G., Dekkers J.F., Bagdany M., Soya N., Xu H., Roldan A., Verkman A.S., Kurth M., Simon A. (2013). Mechanism-Based Corrector Combination Restores Deltaf508-Cftr Folding and Function. Nat. Chem. Biol..

[33] Alshaikh M.K., Tricco A.C., Tashkandi M., Mamdani M., Straus S.E., Ba Hammam A.S. (2012). Sodium Oxybate for Narcolepsy with Cataplexy: Systematic Review and Meta-Analysis. J. Clin. Sleep Med..

[34] Keating G.M. (2014). Sodium Oxybate: A Review of Its Use in Alcohol Withdrawal Syndrome and in the Maintenance of Abstinence in Alcohol Dependence. Clin. Drug Investig..

[35] Kahaly G.J. (2020). Management of Graves Thyroidal and Extrathyroidal Disease: An Update. J. Clin. Endocrinol. Metab..

[36] Truitt C., Hoff W.D., Deole R. (2021). Health Functionalities of Betaine in Patients with Homocystinuria. Front. Nutr..

[37] Kasimer R.N., Langman C.B. (2021). Adult Complications of Nephropathic Cystinosis: A Systematic Review. Pediatr. Nephrol..

[38] Besouw M., Masereeuw R., van den Heuvel L., Levtchenko E. (2013). Cysteamine: An Old Drug with New Potential. Drug Discov. Today.

[39] Devereux G., Steele S., Griffiths K., Devlin E., Fraser-Pitt D., Cotton S., Norrie J., Chrystyn H., O’Neil D. (2016). An Open-Label Investigation of the Pharmacokinetics and Tolerability of Oral Cysteamine in Adults with Cystic Fibrosis. Clin. Drug Investig..

[40] Tosco A., de Gregorio F., Esposito S., de Stefano D., Sana I., Ferrari E., Sepe A., Salvadori L., Buonpensiero P., di Pasqua A. (2016). A Novel Treatment of Cystic Fibrosis Acting on-Target: Cysteamine Plus Epigallocatechin Gallate for the Autophagy-Dependent Rescue of Class Ii-Mutated Cftr. Cell Death Differ..

[41] De Stefano D., Villella V.R., Esposito S., Tosco A., Sepe A., de Gregorio F., Salvadori L., Grassia R., Leone C.A., de Rosa G. (2014). Restoration of Cftr Function in Patients with Cystic Fibrosis Carrying the F508del-Cftr Mutation. Autophagy.

[42] Devereux G., Wrolstad D., Bourke S.J., Daines C.L., Doe S., Dougherty R., Franco R., Innes A., Kopp B.T., Lascano J. (2020). Oral Cysteamine as an Adjunct Treatment in Cystic Fibrosis Pulmonary Exacerbations: An Exploratory Randomized Clinical Trial. PLoS ONE.

[43] Villella V.R., Esposito S., Maiuri M.C., Raia V., Kroemer G., Maiuri L. (2013). Towards a Rational Combination Therapy of Cystic Fibrosis: How Cystamine Restores the Stability of Mutant Cftr. Autophagy.

[44] Ferrari E., Monzani R., Villella V.R., Esposito S., Saluzzo F., Rossin F., D’Eletto M., Tosco A., de Gregorio F., Izzo V. (2017). Cysteamine Re-Establishes the Clearance of Pseudomonas Aeruginosa by Macrophages Bearing the Cystic Fibrosis-Relevant F508del-Cftr Mutation. Cell Death Dis..

[45] Charrier C., Rodger C., Robertson J., Kowalczuk A., Shand N., Fraser-Pitt D., Mercer D., O’Neil D. (2014). Cysteamine (Lynovex(R)), a Novel Mucoactive Antimicrobial & Antibiofilm Agent for the Treatment of Cystic Fibrosis. Orphanet. J. Rare Dis..

[46] Maiuri L., Raia V., Kroemer G. (2017). Strategies for the Etiological Therapy of Cystic Fibrosis. Cell Death Differ..

[47] Tomati V., Caci E., Ferrera L., Pesce E., Sondo E., Cholon D.M., Quinney N.L., Boyles S.E., Armirotti A., Ravazzolo R. (2018). Thymosin Alpha-1 Does Not Correct F508del-Cftr in Cystic Fibrosis Airway Epithelia. JCI Insight.

[48] Armirotti A., Tomati V., Matthes E., Veit G., Cholon D.M., Phuan P.W., Braccia C., Guidone D., Gentzsch M., Lukacs G.L. (2019). Bioactive Thymosin Alpha-1 Does Not Influence F508del-Cftr Maturation and Activity. Sci. Rep..

[49] Rolfe H.M. (2014). A Review of Nicotinamide: Treatment of Skin Diseases and Potential Side Effects. J. Cosmet. Dermatol..

[50] Lin C.C., Hsieh N.K., Liou H.L., Chen H.I. (2012). Niacinamide Mitigated the Acute Lung Injury Induced by Phorbol Myristate Acetate in Isolated Rat’s Lungs. J. Biomed. Sci..

[51] Odera M., Furuta T., Sohma Y., Sakurai M. (2018). Molecular Dynamics Simulation Study on the Structural Instability of the Most Common Cystic Fibrosis-Associated Mutant Deltaf508-Cftr. Biophys. Physicobiol..

[52] Liu K., Watanabe E., Kokubo H. (2017). Exploring the Stability of Ligand Binding Modes to Proteins by Molecular Dynamics Simulations. J. Comput. Aided Mol. Des..

[53] Salentin S., Schreiber S., Haupt V.J., Adasme M.F., Schroeder M. (2015). Plip: Fully Automated Protein-Ligand Interaction Profiler. Nucl. Acids Res..

[54] Prins S., Corradi V., Sheppard D.N., Tieleman D.P., Vergani P. (2022). Can Two Wrongs Make a Right? F508del-Cftr Ion Channel Rescue by Second-Site Mutations in Its Transmembrane Domains. J. Biol. Chem..

[55] Rusnati M., Sala D., Orro A., Bugatti A., Trombetti G., Cichero E., Urbinati C., di Somma M., Millo E., Galietta L.J.V. (2018). Speeding up the Identification of Cystic Fibrosis Transmembrane Conductance Regulator-Targeted Drugs: An Approach Based on Bioinformatics Strategies and Surface Plasmon Resonance. Molecules.

[56] Rusnati M., D’Ursi P., Pedemonte N., Urbinati C., Ford R.C., Cichero E., Uggeri M., Orro A., Fossa P. (2020). Recent Strategic Advances in Cftr Drug Discovery: An Overview. Int. J. Mol. Sci..

[57] Parodi A., Righetti G., Pesce E., Salis A., Tasso B., Urbinati C., Tomati V., Damonte G., Rusnati M., Pedemonte N. (2020). Discovery of Novel Vx-809 Hybrid Derivatives as F508del-Cftr Correctors by Molecular Modeling, Chemical Synthesis and Biological Assays. Eur. J. Med. Chem..

[58] Slater O., Miller B., Kontoyianni M. (2020). Decoding Protein-Protein Interactions: An Overview. Curr. Top. Med. Chem..

[59] Laselva O., Guerra L., Castellani S., Favia M., di Gioia S., Conese M. (2022). Small-Molecule Drugs for Cystic Fibrosis: Where Are We Now?. Pulm. Pharmacol. Ther..

[60] Martelli G., Giacomini D. (2018). Antibacterial and Antioxidant Activities for Natural and Synthetic Dual-Active Compounds. Eur. J. Med. Chem..

[61] Gaskin K.J. (2013). Nutritional Care in Children with Cystic Fibrosis: Are Our Patients Becoming Better?. Eur. J. Clin. Nutr..

[62] Yang H., Ma T. (2015). F508del-Cystic Fibrosis Transmembrane Regulator Correctors for Treatment of Cystic Fibrosis: A Patent Review. Expert Opin. Ther. Patents.

[63] Dey I., Shah K., Bradbury N.A. (2016). Natural Compounds as Therapeutic Agents in the Treatment Cystic Fibrosis. J. Genet. Syndr. Gene Ther..

[64] Esposito S., Testa I., Zani E.M., Cunico D., Torelli L., Grandinetti R., Fainardi V., Pisi G., Principi N. (2022). Probiotics Administration in Cystic Fibrosis: What Is the Evidence?. Nutrients.

[65] Gur M., Zuckerman-Levin N., Masarweh K., Hanna M., Laghi L., Marazzato M., Levanon S., Hakim F., Bar-Yoseph R., Wilschanski M. (2022). The Effect of Probiotic Administration on Metabolomics and Glucose Metabolism in Cf Patients. Pediatr. Pulmonol..

[66] Scambi C., de Franceschi L., Guarini P., Poli F., Siciliano A., Pattini P., Biondani A., la Verde V., Bortolami O., Turrini F. (2009). Preliminary Evidence for Cell Membrane Amelioration in Children with Cystic Fibrosis by 5-Mthf and Vitamin B12 Supplementation: A Single Arm Trial. PLoS ONE.

[67] Nowak J.K., Krzyzanowska-Jankowska P., Drzymala-Czyz S., Gozdzik-Spychalska J., Wojsyk-Banaszak I., Skorupa W., Sapiejka E., Miskiewicz-Chotnicka A., Brylak J., Zielinska-Psuja B. (2022). Fat-Soluble Vitamins in Standard vs. Liposomal Form Enriched with Vitamin K2 in Cystic Fibrosis: A Randomized Multi-Center Trial. J. Clin. Med..

[68] Turowski J.B., Pietrofesa R.A., Lawson J.A., Christofidou-Solomidou M., Hadjiliadis D. (2015). Flaxseed Modulates Inflammatory and Oxidative Stress Biomarkers in Cystic Fibrosis: A Pilot Study. BMC Complement. Altern. Med..

[69] Soares V.E.M., do Carmo T.I.T., Anjos F.D., Wruck J., de Oliveira Maciel S.F.V., Bagatini M.D., de Resende E.S.D.T. (2022). Role of Inflammation and Oxidative Stress in Tissue Damage Associated with Cystic Fibrosis: Cape as a Future Therapeutic Strategy. Mol. Cell Biochem..

[70] Carbone A., Montalbano A., Spano V., Musante I., Galietta L.J.V., Barraja P. (2019). Furocoumarins as Multi-Target Agents in the Treatment of Cystic Fibrosis. Eur. J. Med. Chem..

[71] Sohma Y., Yu Y.C., Hwang T.C. (2013). Curcumin and Genistein: The Combined Effects on Disease-Associated Cftr Mutants and Their Clinical Implications. Curr. Pharm. Des..

[72] Lord R., Fairbourn N., Mylavarapu C., Dbeis A., Bowman T., Chandrashekar A., Banayat T., Hodges C.A., Al-Nakkash L. (2018). Consuming Genistein Improves Survival Rates in the Absence of Laxative in Deltaf508-Cf Female Mice. Nutrients.

[73] Wegrzyn G., Jakobkiewicz-Banecka J., Gabig-Ciminska M., Piotrowska E., Narajczyk M., Kloska A., Malinowska M., Dziedzic D., Golebiewska I., Moskot M. (2010). Genistein: A Natural Isoflavone with a Potential for Treatment of Genetic Diseases. Biochem. Soc. Trans..

[74] Han M.K., Barreto T.A., Martinez F.J., Comstock A.T., Sajjan U.S. (2020). Randomised Clinical Trial to Determine the Safety of Quercetin Supplementation in Patients with Chronic Obstructive Pulmonary Disease. BMJ Open Respir. Res..

[75] Belchamber K.B.R., Donnelly L.E. (2020). Targeting Defective Pulmonary Innate Immunity-A New Therapeutic Option?. Pharmacol. Ther..

[76] Moran O., Galietta L.J., Zegarra-Moran O. (2005). Binding Site of Activators of the Cystic Fibrosis Transmembrane Conductance Regulator in the Nucleotide Binding Domains. Cell Mol. Life Sci..

[77] Yu B., Zhang Y., Sui Y., Yang S., Luan J., Wang X., Ma T., Yang H. (2013). Potentiation of Mutant Cftr Cl- Channel Currents by the Naturally Occurring Stilbene Compound Resveratrol. Pharmazie.

[78] Pyle L.C., Fulton J.C., Sloane P.A., Backer K., Mazur M., Prasain J., Barnes S., Clancy J.P., Rowe S.M. (2010). Activation of the Cystic Fibrosis Transmembrane Conductance Regulator by the Flavonoid Quercetin: Potential Use as a Biomarker of Deltaf508 Cystic Fibrosis Transmembrane Conductance Regulator Rescue. Am. J. Respir. Cell Mol. Biol..

[79] Centko R.M., Carlile G.W., Barne I., Patrick B.O., Blagojevic P., Thomas D.Y., Andersen R.J. (2020). Combination of Selective Parp3 and Parp16 Inhibitory Analogues of Latonduine a Corrects F508del-Cftr Trafficking. ACS Omega.

[80] Dekkers J.F., van Mourik P., Vonk A.M., Kruisselbrink E., Berkers G., Groot K.M.de., Janssens H.M., Bronsveld I., van der Ent C.K., de Jonge H.R. (2016). Potentiator Synergy in Rectal Organoids Carrying S1251n, G551d, or F508del Cftr Mutations. J. Cyst. Fibros..

[81] Wang W., Hong J.S., Rab A., Sorscher E.J., Kirk K.L. (2016). Robust Stimulation of W1282x-Cftr Channel Activity by a Combination of Allosteric Modulators. PLoS ONE.

[82] Cho D.Y., Zhang S., Lazrak A., Grayson J.W., Garcia J.A.P., Skinner D.F., Lim D.J., Mackey C., Banks C., Matalon S. (2019). Resveratrol and Ivacaftor Are Additive G551d Cftr-Channel Potentiators: Therapeutic Implications for Cystic Fibrosis Sinus Disease. Int. Forum Allergy Rhinol..

[83] Pesce E., Gorrieri G., Sirci F., Napolitano F., Carrella D., Caci E., Tomati V., Zegarra-Moran O., di Bernardo D., Galietta L.J. (2016). Evaluation of a Systems Biology Approach to Identify Pharmacological Correctors of the Mutant Cftr Chloride Channel. J. Cyst. Fibros..

[84] Huang B., Zhang Y. (2022). Teaching an Old Dog New Tricks: Drug Discovery by Repositioning Natural Products and Their Derivatives. Drug Discov. Today.

[85] Fernandes C.A., Fievez L., Ucakar B., Neyrinck A.M., Fillee C., Huaux F., Delzenne N.M., Bureau F., Vanbever R. (2011). Nicotinamide Enhances Apoptosis of G(M)-Csf-Treated Neutrophils and Attenuates Endotoxin-Induced Airway Inflammation in Mice. Am. J. Physiol. Lung Cell Mol. Physiol..

[86] Stutts M.J., Gabriel S.E., Price E.M., Sarkadi B., Olsen J.C., Boucher R.C. (1994). Pyridine Nucleotide Redox Potential Modulates Cystic Fibrosis Transmembrane Conductance Regulator Cl- Conductance. J. Biol. Chem..

[87] Mendoza J.L., Schmidt A., Li Q., Nuvaga E., Barrett T., Bridges R.J., Feranchak A.P., Brautigam C.A., Thomas P.J. (2012). Requirements for Efficient Correction of Deltaf508 Cftr Revealed by Analyses of Evolved Sequences. Cell.

[88] Hwang E.S., Song S.B. (2020). Possible Adverse Effects of High-Dose Nicotinamide: Mechanisms and Safety Assessment. Biomolecules.

[89] Wang F., Zeltwanger S., Yang I.C., Nairn A.C., Hwang T.C. (1998). Actions of Genistein on Cystic Fibrosis Transmembrane Conductance Regulator Channel Gating. Evidence for Two Binding Sites with Opposite Effects. J. Gen. Physiol..

[90] Laselva O., Qureshi Z., Zeng Z.W., Petrotchenko E.V., Ramjeesingh M., Hamilton C.M., Huan L.J., Borchers C.H., Pomes R., Young R. (2021). Identification of Binding Sites for Ivacaftor on the Cystic Fibrosis Transmembrane Conductance Regulator. iScience.

[91] Shaughnessy C.A., Zeitlin P.L., Bratcher P.E. (2021). Elexacaftor Is a Cftr Potentiator and Acts Synergistically with Ivacaftor During Acute and Chronic Treatment. Sci. Rep..

[92] Trott O., Olson A.J. (2010). Autodock Vina: Improving the Speed and Accuracy of Docking with a New Scoring Function, Efficient Optimization, and Multithreading. J. Comput. Chem..

[93] Gasteiger J., Marsili M. (1978). A new model for calculating atomic charges in molecules. Tetrahedron.

[94] Case D.A., Cheatham T.E., TDarden, Gohlke H., Luo R., Merz K.M., Onufriev A., Simmerling C., Wang B., Woods R.J. (2005). The Amber Biomolecular Simulation Programs. J. Comput. Chem..

[95] Maier J.A., Martinez C., Kasavajhala K., Wickstrom L., Hauser K.E., Simmerling C. (2015). Ff14sb: Improving the Accuracy of Protein Side Chain and Backbone Parameters from Ff99sb. J. Chem. Theory Comput..

[96] Dickson C.J., Madej B.D., Skjevik A.A., Betz R.M., Teigen K., Gould I.R., Walker R.C. (2014). Lipid14: The Amber Lipid Force Field. J. Chem. Theory Comput..

[97] O’Ryan L., Rimington T., Cant N., Ford R.C. (2012). Expression and Purification of the Cystic Fibrosis Transmembrane Conductance Regulator Protein in Saccharomyces Cerevisiae. J. Vis. Exp..

[98] Pollock N., Cant N., Rimington T., Ford R.C. (2014). Purification of the Cystic Fibrosis Transmembrane Conductance Regulator Protein Expressed in Saccharomyces Cerevisiae. J. Vis. Exp..

[99] Trutnau H.H. (2006). New Multi-Step Kinetics Using Common Affinity Biosensors Saves Time and Sample at Full Access to Kinetics and Concentration. J. Biotechnol..

[100] Pedemonte N., Bertozzi F., Caci E., Sorana F., di Fruscia P., Tomati V., Ferrera L., Rodriguez-Gimeno A., Berti F., Pesce E. (2020). Discovery of a Picomolar Potency Pharmacological Corrector of the Mutant Cftr Chloride Channel. Sci. Adv..

[101] Pedemonte N., Lukacs G.L., Du K., Caci E., Zegarra-Moran O., Galietta L.J., Verkman A.S. (2005). Small-Molecule Correctors of Defective Deltaf508-Cftr Cellular Processing Identified by High-Throughput Screening. J. Clin. Investig..

[102] Sondo E., Tomati V., Caci E., Esposito A.I., Pfeffer U., Pedemonte N., Galietta L.J. (2011). Rescue of the Mutant Cftr Chloride Channel by Pharmacological Correctors and Low Temperature Analyzed by Gene Expression Profiling. Am. J. Physiol. Cell Physiol..

[103] Sondo E., Falchi F., Caci E., Ferrera L., Giacomini E., Pesce E., Tomati V., Bertozzi S.M., Goldoni L., Armirotti A. (2018). Pharmacological Inhibition of the Ubiquitin Ligase Rnf5 Rescues F508del-Cftr in Cystic Fibrosis Airway Epithelia. Cell Chem. Biol..

