# Supplementary material for: Virtual Drug Repositioning as a Tool to Identify Natural Small Molecules That Synergize with Lumacaftor in F508del-CFTR Binding and Rescuing
Source: Int J Mol Sci. 2022 Oct 14;23(20):12274. doi: 10.3390/ijms232012274 (PMC9602983; doi:10.3390/ijms232012274)
Supplement: Supplementary file 1 [file ijms-23-12274-s001.zip › ijms-1908455-supplementary.pdf]

Supporting Information

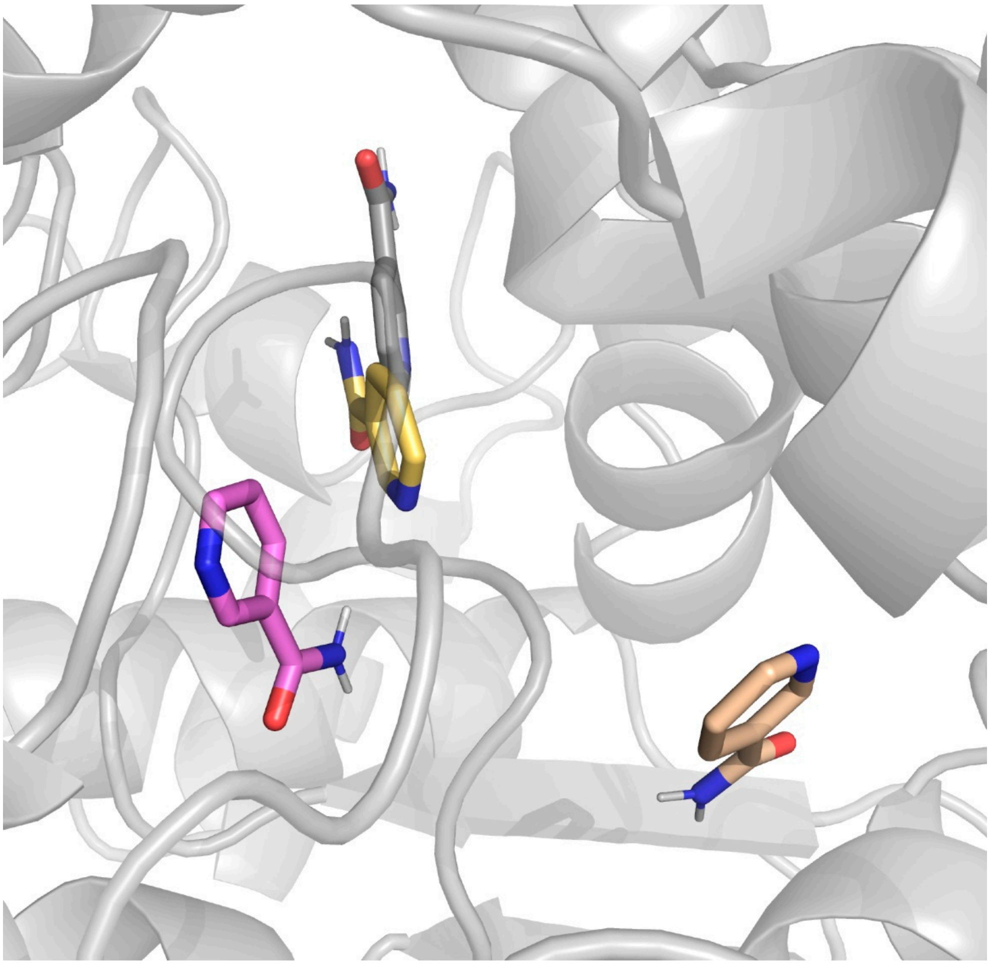

Figure S1. MD replicas results of NAM alone inside the DP1 sub-pocket of F508del-CFTR. The protein is represented in cartoon. The docking pose of NAM is represented in grey stick, while in purple, yellow, and brown are represented the ending position of NAM after 50 ns MD replicas, respectively.
